# Predictive Value of *CD44* for Prognosis in Patients with Breast Cancer

**DOI:** 10.31557/APJCP.2020.21.9.2561

**Published:** 2020-09

**Authors:** Yousef Roosta, Zohreh Sanaat, Ali Reza Nikanfar, Roya Dolatkhah, Ashraf Fakhrjou

**Affiliations:** *Hematology and Oncology Research Center, Tabriz University of Medical Sciences, Tabriz, Iran. *

**Keywords:** CD44, clinicopathological factors, breast cancer, HER2, Prognostic Factor

## Abstract

**Background:**

Breast Cancer (BC), is one of the most common malignancies around the world.* CD44* expression correlates with cell proliferation, infiltration, angiogenesis, metastasis and prognosis in breast cancer but the exact mechanism of CD44 function is still not clear. The present study evaluates the expression of *CD44* in primary HER2-positive breast cancer. The results can be used to determine the disease-free and overall survival of patients with breast cancer.

**Methods:**

We studied specimens from 100 patients with HER2-positive invasive breast cancer between March 2011 and June 2019. Immunohistochemical staining for CD44 was performed in all the specimens. Their CD44 association with clinicopathologic parameters and prognosis was evaluated.

**Results:**

The high *CD44* was expression in 68(68%) of the patients and Low expression in 32(32%). *CD44* expression was significantly associated with stage (p=0.007). There were no significant associations between the DFS, OS and other clinicopathologic parameters except for the stage, respectively (HR= 3.67, 95% CI =1.16-11.56, P = 0.03) (HR= 0.8.56, 95% CI =2.22-32.90, P = 0.002).20% of patients had died by the end of the follow-up. There were no significant association between *DFS*, *OS* and *CD44 *expression, respectively. (Log-rank p=0.13). (Log-rank p=0.10).

**Conclusion:**

The results from this study suggest that CD44 is clinically associated with stage of breast cancers. From the survival analysis, there was no statistical difference in overall survival and disease free survival with respect to *CD44* expression. Further studies larger sample sizes are recommended for further investigation.

## Introduction

The beginning of the 21^st^ century has been marked by intensified research on molecular genetics, epigenetics and the metabolic factors of cancer progression and treatment of patients (Chekhun et al, 2017).Breast cancer is now ranked the second most common malignancy(Jamel et al, 2010) ,the accounts for about 1.38 million new cases diagnosed every year (Ferlay et al, 2010). 

 Breast cancer is a highly heterogeneous disease with distinct biological and clinical behaviors and responses to treatment that can be classified into different subtypes based on histopathology type as well as molecular profile (Zhang et al., 2012; Hu et al., 2006).

 Approximately 20-30% of breast cancer cases are HER2-positive owing to the over-expression and/or amplification of the *HER2 *gene (Dunnwald et al., 2007; Moasser et al., 2007). HER2-positivity is associated with aggressive clinical behaviors and poor clinical outcomes (Campone et al., 2011; Elster et al., 2015; Wange et al., 2003, Wange et al., 2012; Slamon et al., 1989).

Evidence suggests that some possible mechanisms for resistance to trastuzumab have been Cancer Stem Cells (CSCs), with ability to self-renew and differentiate and interfere with metastasis and contribute to chemo resistance and thus lead to tumor recurrence and relapse (Salmon et al., 1987; Rimawi et al., 2015). Recent observations showed that treatment with trastuzumab may directly affect BCSCs in HER2-positive breast cancer (Petrelli et al., 2012 ;Guo et al., 2015). In contrast, resistance to trastuzumab may be guided by BCSC (Petrelli et al., 2012; Guo et al., 2015). In 2003, Al-Hajj showed that tumorigenic breast cancer cells significantly exhibited stem cell-like properties, such as CD44+/CD24/low (Boulbes et al., 2015). The clinicopathological and prognostic significance of these findings is still controversial (Esteva, 2004; Swain et al., 2013). 

CD44 is a Trans membrane glycoprotein molecule (gene is located on the chromosome 11p13) is widely expressed in the epithelial, mesenchymal and hematopoietic cells. Also, is involved in, division, survival, migration and adhesion of cells (Bedard et al., 2009). 

A correlation has also been reported between *CD44 *expression and Breast Cancer (BC) cell proliferation, infiltration, angiogenesis, metastasis and prognosis (Seo et al., 2016). Several studies have examined the relationship between CSCs and resistance to trastuzumab, resistance is indirectly guided by CSCs (Clarke and Fuller, 2006; Polyak and Hahn, 2006). Considering the importance of CSC markers for breast cancer and the recent evidence on HER2-positive breast cancer being treated with trastuzumab, which directly targets CSC. Although CD44 and HER2 are both negative predictors, it is still unclear whether they are related to each other. The present study thus evaluates the expression of *CD44* as a surrogate marker for BCSCs by immunohistochemistry in primary HER2-positive breast cancer. It further examines the association of *BCSC* marker expression with the clinicopathological significance and prognostic value of HER2 positivity in breast cancer treated with trastuzumab. The results can be used to determine the disease-free and overall survival of patients with breast cancer.

## Materials and Methods

*Patients and Samples*

This study was approved by the ethics committee of Tabriz University of Medical Sciences (under the code 5/d/584008) and all the patients signed an informed consent form at the time of referring to the study clinic which permitted the review of their medical records and tissue sample slides for future studies. The paraffin-embedded tumor tissues of 100 patients with invasive ductal carcinoma were available between March 2011 and June 2019 was analyzed in Tabriz University of Medical Sciences.

The archived slides of breast tumor tissue stained with hematoxylin and eosin were retrieved for all the cases and reviewed to confirm their pathological features based on the 2012 WHO classification (Kakarala and Wicha, 2008) .The suitable tissue blocks were identified for immunohistochemical (IHC) analysis.

The patients’ medical records were reviewed for gathering their clinical data, including age at diagnosis, menopausal status, type of surgery, tumor size (T), histologic grade, axillary lymph node status (N),metastases status(M) ,tumor stage, lymphatic and neural and vascular invasion, estrogen receptor (ER) status, progesterone receptor (PR) status, P53, Ki-67, HER2 status and tumor recurrence or distant metastasis. All the patients received routine chemotherapy, endocrine and trastuzumab therapy following their surgery. Pathologic TNM classifica¬tion and staging were also performed for all the cases using the seventh edition of the American Joint Committee on Cancer Criteria (Li et al., 2008).

*Follow-up*

The overall survival (OS, in months) was measured based on all the occurred deaths regardless of their cause. Disease-free survival (DFS, in months) was defined as the time relapsed between the excisions of the primary tumor to the manifesta¬tion of local or distant metastasis.

*Immunohistochemistry Analysis*

**Figure 1:**
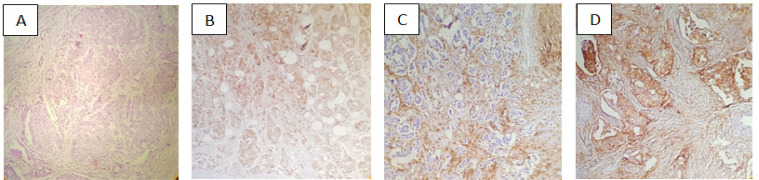
Immunohistochemical Analysis of CD44 in Breast Cancer(x100). Note : (A) Hemotoxylin and Eosin stain, (B) HER2 +3, (C) CD44 negative, (D) CD44 positive

IHC staining was performed in all the cases for ER, PR, HER2, Ki-67, P53 and CD44 biomarkers. For the histologic study, tumor samples were fixed in buffered formalin 10% and embedded as paraffin blocks. The sections were made from the formalin-fixed, paraffin-embedded blocks in four micrometers and were then deparaffinized in xylene and rehydrated in a graded series of alcohol 96% and 100% solutions and then cooked with EDTA buffer at a pH of 6.0 at a sub-boiling temperature in three steps (each step taking 5 min) and ultimately cooled for 20 minutes at room temperature. The sections were washed twice with Tris-Buffered Saline (TBS) for 10 min. The sections were incubated in H_2_O_2_ 3% in methanol for 10 min to avoid tissue destruction. After the overnight incubation of CD44 ready to use antibody in 4°C refrigerator, the sections were detected with envision and chromogen. Afterwards, the slides were counterstained with Hematoxylin, dehydrated and then mounted. The following primary antibodies were used: ER (clone ID5; Dako Denmark A/S, Glostrup, Denmark), PR (clone PgR636; Dako Denmark A/S), Ki-67 (clone MIB-1; Dako Denmark A/S), P53 (cloneD07; Dako Denmark A/S), HER/2neu (REF: A0485, 1/200; Dako Denmark A/S) and CD44 (Clone: 156-3C11; HCAM, Diagnostic BioSystem, Pleasanton, CA, USA).The Haematoxylin and Eosin (H&E) stain of breast sample and IHC stain for HER2 , +3 Shown in (Figure 1 A ,B). The Haematoxylin and Eosin (H&E) stain of breast sample and IHC stain for HER2, +3 Shown in (Figure 1 A, B). CD44 immunostaining score was incorporated both staining intensity (0=absent, 1=weak, 2=moderate, 3=strong) and percentage of positive cells (0=0%, 1=1-25%, 2=26-50%, 3=51-75%, 4=76-100% of cells). The immunostaining score was calculated based on the proportion of stained tumor cells: 0-10% as negative (-), 11-25% as slightly positive (+), 26-50% as moderately positive (++), and 51-100% as strongly positive (+++). Patients with - and + expression was combined as the lower expression group, and patients with ++ and +++ expression was combined as the higher expression group for analyses (Tanei et al, 2009) (Figure 1 C, D).

*Statistical analysis*

*CD44* expression was considered as binary independent factor (low and high level of CD44). The association between *CD44* expressions and clinicopathologic parameters was examined with Chi-square test or Fisher’s exact test. The Kaplan-Meier method was used to evaluate survival rates and log rank test was used to compare survival of patients with low and high level of CD44. The univariate Cox proportional hazard regression model was performed to observe the association of CD44 with survival rates. The multivariate Cox proportional hazard regression analysis was performed to assess association of CD44 with OS and PFS, adjusted for confounding variables (grade, tumor size, axillary node, metastases, stage, *ER*,* PR* and *CD44* expression. The proportional hazard assumption was checked for Cox regression based on Schoenfeld residuals. Harrell’s C-index was calculated to determine the predictive power of CD44 by Survival rates. The p-value < 0.05 were considered significant. All the statistical analyses were performed in STATA, Version 14.

## Results

*Clinicopathological Features*

The mean±SD age of the patients was 46.94±9.63 years (range: 26–80 years). About 67% of cases were younger than 50 years old. 

*Association between clinicopathologic parameters and CD44 expression*

The high *CD44* was expression in 68(68%) of the patients and Low expression in 32(32%). *CD44* expression was significantly associated with stage (p=0.007). There were no relationships between *CD44* expression and other clinicopathologic parameters (Table 1).

*Survival analysis based on CD44 expression*

**Figure 2:**
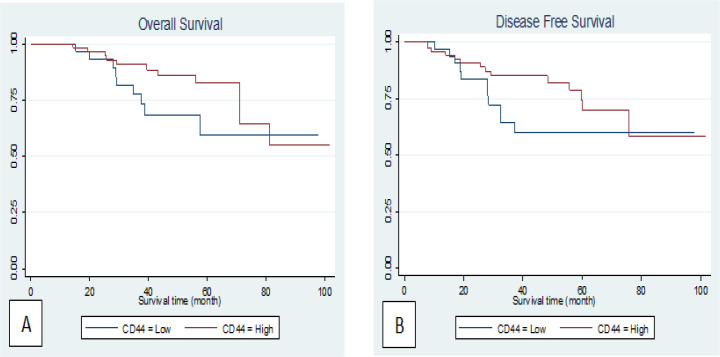
Kaplan–Meier Curves Showing association of CD44 Expression in Patients with Breast Cancer. (A) Overall Survival (P=0.10) and (B), Disease-Free Survival (P=0.13)

Survival analysis was estimated using the Kaplan-Meier method. 20% of patients had died by the end of the follow-up. In 75% of quadrants of, on average 71% of patients were alive There were no significant association between *OS* and *CD44* expression (log-rank p=0.10; Figure 2A). 5 patients (of the 80 live breast cancers) had relapsed by the end of the follow-up. In 75% of quadrants, on average 48.4% of patients were alive without any relapse. There were no significant association between *DFS* and *CD44* expression (log-rank p=0.13; Figure 2B). 

*Association between patient outcome and CD44 expression*

The Univariate Cox regression analysis showed that a poor DFS was not association with *CD44* expression (HR=0.5, 95% CI=0.25-1.20, p=.14). The proportion hazard assumption was satisfied for DFS (p-value=0.44). Harrell’s-C index for DFS was 0.58. Also, the univariate Cox regression analysis showed that OS was not association with *CD44* expression (HR=0.48, 95% CI =0.20-1.18, p=0.11). The proportion hazard assumption was satisfied for OS (p-value=0.50).

*Harrell’s-C index for OS was 0.60*

After adjustment for other clinicopathologic parameters, cox-regression analysis showed that there were no significant associations between the DFS and *CD44* expression (HR= 0.78, 95% CI= 0.30-1.04, P = 0.61). Cox-regression analysis also revealed that a poor OS was not significantly associated with CD44 (HR =0.85, 95% CI= 0.29-2.50, P = 0.77). There were no significant associations between the DFS, OS and other clinicopathologic parameters except for the stage, respectively (HR= 3.67, 95% CI =1.16-11.56, P = 0.03) (HR= 0.8.56, 95% CI =2.22-32.90, P = 0.002). (Table 2) Neural, vascular, and lymphatic variables were omitted from multivariate analysis of OS and DFS due to the Spars and over-estimation. 

**Table 1:** Clinicopathologic characteristics of patients with breast cancer according to the CD44 level

Variables	Total	CD44		P value
		Low (%)	High (%)	
Age				
< 50	67	22 (32.8)	45 (67.2)	0.8
>=50	33	10 (30.3)	23 (69.7)	
Grade				
I	14	4 (28.60	10 (71.4)	1*
II	82	27 (32.9)	55 (67.1)	
III	4	1 (250	3 (75)	
T				
<=2	31	7 (22.6)	24 (77.4)	0.24
2-5	59	20 (33.9)	39 (66.1)	
>5	10	5 (50)	5 (50)	
N				
Positive	73	28 (38.4)	45 (61.6)	0.25
Negative	27	4 (14.8)	23 (85.2)	
M				
Positive	7	3 (42.9)	4 (57.1)	0.67*
Negative	93	29 (31.2)	64 (68.8)	
Stage				
I,II	71	17 (23.9)	54 (76.1)	0.007
III,IV	29	15 (51.7)	14 (48.3)	
Lymphatic Invasion				
Positive	81	28 (34.6)	53 (65.4)	0.25
Negative	19	4 (21.2)	15 (78.9)	
Neural Invasion				
Positive	74	25 (33.8)	49 (66.20	0.51
Negative	26	7 (26.9)	19 (73.1)	
Vascular Invasion				
Positive	86	28 (32.6)	58 (67.4)	0.76
Negative	14	4 (28.6)	10 (71.4)	
ER				
Positive	96	24 (34.8)	45 (65.2)	0.4
Negative	31	8 (25.8)	23 (74.2)	
PR				
Positive	64	22 (34.4)	42 (65.6)	0.5
Negative	36	10 (27.8)	26 (72.2)	
P 53				
Positive	51	16 (31.4)	35 (68.6)	0.9
Negative	49	16 (32.7)	33 (67.3)	
Ki 67				
Positive	64	22 (34.4)	42 (65.6)	0.5
Negative	36	10 (27.8)	26 (72.2)	
DFS				
Alive	75	21 (28)	54 (72)	0.13
Relapse	25	11 (44)	14 (56)	
OS				
Alive	80	23 (28.8)	57 (71.3)	0.2
Death	20	9 (45)	11 (55)	

*Fisher’s exact test; ER, estrogen receptor; PR, progesterone receptor, DFS, Disease Free Survival, OS, Overall Survival.

**Table 2:** Hazard Ratios of CD44 for DFS and OS Adjusted for Clinicopathologic Characteristics of Patients with Breast Cancer (Multivariate Cox Proportional-Hazards Regression Model)

Variables		DFS	*P*-value	OS	*P*-value
		HR (95% CI)		HR (95% CI)	
Grade	I	Ref			
	II	3.31 (0.41-26.50)	0.25	2.73 (0.32-23.16)	0.35
	III	4.90 (0.23-101.72)	0.3	4.60 (0.20-106.52)	0.34
T	<=2	Ref			
	2-5	0.40 (0.15-1.13)	0.08	0.55 (0.18-1.74)	0.31
	>5	0.52 (0.11-2.40)	0.4	0.20 (0.03-1.17)	0.07
N	Positive	Ref			
	Negative	0.65 (0.19-2.20)	0.5	0.94 (0.22-3.40)	0.93
M	Positive	Ref			
	Negative	0.31 (0.09-1.09)	0.07	0.75 (0.20-2.88)	0.7
Stage	I,II	Ref			
	III,IV	3.70 (1.16-11.60)	0.03	8.60 (2.22-32.90)	0.002
ER	Positive	Ref			
	Negative	0.80 (0.13-4.61)	0.8	0.28 (0.04-1.95)	0.2
PR	Positive	Ref			
	Negative	2.51 (0.50-13.54)	0.3	4.08 (0.70-24.62)	0.12
CD 44	Low	Ref			
	High	0.80 (0.30-2.04)	0.61	0.85 (0.30-2.50)	0.77

Notes: Lower, lower bound for 95% CI; Upper, upper bound for 95% CI; adjusted variables: Grade, Tumor size, Lymph node status, Metastases, Stage, ER, PR expression; ER, estrogen receptor; PR, progesterone receptor; DFS, disease free survival; OS, overall survival

## Discussion

Breast carcinoma is the leading cause of cancer death in women and is one of the most common malignancies in the world (Jemal et al., 2010). Breast cancer stem cells are a small population of cells that have the classic features of cancer stem cells and are transformed by the accumulation of mutations in the tumor cells (Jemalet al., 2010).The initial detection of breast cancer stem cells takes place based on the observation of a combination of CD44 and CD24 (Boulbes et al., 2015).

In this study, high *CD44* was expression in 68(68%) of the patients and Low expression in 32 (32%). *CD44 *expression was significantly associated with stage (p=0.007). There were no relationships between* CD44 *expression and other clinicopathologic parameters. Since Al-Hajj et al., (2003) revealed for the first time that the tumorigenic stem cells in breast cancer have the CD44+, the tumorigenic potential and invasive features of this phenotype have been confirmed repeatedly (Mylona et al., 2008; Abraham et al., 2005) Olsson et al., (2011), Sanchez et al., (2001), Found that there are no significant associations between the *CD44* expression and tumor size, lymph node status and hormone receptor. In our study we also did not find this association.According to the results obtained by Horiguchi et al., (2010), a higher expression of *CD44* is significantly associated with a smaller tumor size, lack of axillary lymph node involvement and lower stages of breast cancer. In our study CD44 was associated just with higher stages. Bânkfalvi et al., (1998), observed that increased *CD44 *expression is associated with lymph node involvement in breast cancer. Looi et al., (2006), showed that CD44 plays an important role in the progression of breast cancer, *CD44* expression increased in cases of breast cancer, which is associated with a high-grade tumor. These results contradict the present findings. Based on these disparate findings, there are probably several factors that may affect the expression of these markers in human breast cancer, including the use of different antibodies and conditions for immunohistochemistry (Olsson et al, 2011; Resetkova et al, 2010; Almed et al, 2012) CSCs are responsible for the initiation, progression and recurrence of various types of cancers, including breast cancer; however, we didn’t find any significant association between *CD44* expression and clinical outcomes in this study. There were no significant differences between OS, *DFS* and *CD44* expression (log-rank p=0.10; Figure 2A; log-rank p=0.13; Figure 2B).

Abraham et al., (2005), reported no significant relationships between the CD44+ phenotype and survival in patients with breast cancer, which is similar to the present findings. Other studies, such as those by (Mylona et al., 2008; Shipitsin et al., 2007; Kim et al., 2011; McFarlane et al., 2015), have suggested that *CD44 *expression is associated with a poor prognosis and can be considered a target for the treatment of breast cancer. The findings of those study and other studies cannot clearly elaborate the role of CSC in tumor.

In the present study, a poor DFS and OS was not association with* CD44 *expression, respectively. (HR=0.5, 95% CI=0.25-1.20, p=.14), (HR =0.85, 95% CI= 0.29-2.50, P = 0.77).

The limitations of this research include the study of invasive breast cancer cases only. Camerlingo et al., (2014), and de Beca et al., (2013), examined the expression of *CSC* in various histological subtypes of breast cancer. Camerlingo did not report any association between histological subtype and the CD44 + phenotype, while De Beca et al., (2013) reported that modular, papillary and tubular carcinoma are expressed in the CD44+ phenotype. Second limitations of this research include the need for more patients with all breast cancer subtypes; third, the use of different types of antibodies and diverse conditions for immunohistochemistry; forth, not performing CD24 for economic reasons and evaluating CD44 only in the HER2-positive patients. 

To the researchers’ knowledge, the present research is the first effort in Iran to examine possible associations between CD44 and clinicopathological features and outcomes in HER2-positive breast cancer patients. The results showed that no significant associations were found between *CD44* expression and clinical outcomes in this study. Future studies need to verify these results using larger sample sizes and to investigate the association between CSC and clinicopathological features and prognostic parameters and their role in the treatment of breast cancer.
